# Effects of Substrate Bias Voltage on Structure of Diamond-Like Carbon Films on AISI 316L Stainless Steel: A Molecular Dynamics Simulation Study

**DOI:** 10.3390/ma14174925

**Published:** 2021-08-30

**Authors:** Ngoc-Tu Do, Van-Hai Dinh, Le Van Lich, Hong-Hue Dang-Thi, Trong-Giang Nguyen

**Affiliations:** 1 Department of Industrial Equipment & Tools, Faculty of Mechanical Engineering, Hanoi University of Industry, Hanoi 100000, Vietnam; tudn@haui.edu.vn; 2 School of Materials Science and Engineering, Hanoi University of Science and Technology, Hanoi 100000, Vietnam; hue.dangthihong@hust.edu.vn (H.-H.D.-T.); giang.nguyentrong@hust.edu.vn (T.-G.N.)

**Keywords:** diamond-like carbon, substrate bias voltage, AISI 316L stainless steel, deposition process, molecular dynamics simulation

## Abstract

With the recent significant advances in micro- and nanoscale fabrication techniques, deposition of diamond-like carbon films on stainless steel substrates has been experimentally achieved. However, the underlying mechanism for the formation of film microstructures has remained elusive. In this study, the growth processes of diamond-like carbon films on AISI 316L substrate are studied via the molecular dynamics method. Effects of substrate bias voltage on the structure properties and sp^3^ hybridization ratio are investigated. A diamond-like carbon film with a compact structure and smooth surface is obtained at 120 V bias voltage. Looser structures with high surface roughness are observed in films deposited under bias voltages of 0 V or 300 V. In addition, sp^3^ fraction increases with increasing substrate bias voltage from 0 V to 120 V, while an opposite trend is obtained when the bias voltage is further increased from 120 V to 300 V. The highest magnitude of sp^3^ fraction was about 48.5% at 120 V bias voltage. The dependence of sp^3^ fraction in carbon films on the substrate bias voltage achieves a high consistency within the experiment results. The mechanism for the dependence of diamond-like carbon structures on the substrate bias voltage is discussed as well.

## 1. Introduction

Stainless steel alloys have been widely used in orthopedic implants [1,2] and various industrial areas [3] because of their desirable mechanical properties, non-toxicity, biological safety, and cost effectiveness. However, these alloys exhibit some poor properties such as weak chemical bonds with natural bones [4], low hardness, and low corrosion resistance [3]. To overcome these disadvantages, surface engineering technologies are commonly applied to improve the surface properties of stainless steels through various coatings and biofunctionalization methods [4,5,6]. Several coating films have been employed for surface treatments of stainless steels, including carbon-based films, noble metals, metal nitrides, and polymers [7,8,9,10]. Recently, diamond-like carbon (DLC) coatings have attracted a lot of attention due to their uniquely combined properties, such as high hardness, low friction and wear, electrical insulation, and chemical inertness, and, thereby, have become a potential protective coating in corrosion media [11,12]. These superior properties originate from the unique DLC structure, where clusters of graphite structure with C-C sp^2^ bonding interconnect with a random network of diamond structure with sp^3^ bonding, facilitating a synergistic effect of these structures. Properties of DLC films, thus, significantly depend on the sp^3^/sp^2^ hybridization ratio. On the other hand, when depositing DLC films on stainless steel substrates, the films present low adhesion strength and low sp^3^ hybridization fraction [13,14]. Therefore, property enhancement of DLC film deposited on stainless steels is necessary.

Experimentally, numerous studies have been conducted to control the sp^2^ and sp^3^ hybridization fractions in DLC films deposited on stainless steel substrates using various methods such as sputtering, plasma immersion ion plantation, plasma enhanced chemical vapor deposition, ion plating, and filtered cathodic arc [15]. Jin et al. [16] investigated the effect of substrate bias voltage on the structural properties of DLC films deposited on 304 stainless steel substrates through closed unbalanced magnetron sputtering. The obtained results indicated that higher bias voltage promotes sp^2^ hybridization. Bi et al. [17] later used closed unbalanced magnetron sputtering to investigate the effects of substrate bias voltage on the structural properties of DLC films deposited on 316L stainless steel samples. Sp^3^ fraction in DLC films can be achieved a high value of about 50% at a moderate bias voltage of 120 V. Yi et al. [18] have studied microstructures and properties of DLC films deposited on stainless steel plates under different argon flow rates via closed unbalanced magnetron sputtering. This study showed that the sp^3^ fraction in DLC film is low at a moderate argon flow rate around 50 sccm, while it is higher in the other ranges of argon flow rate. Dong et al. [19] investigated the effect of temperature on microstructures of DLC films on 304 stainless steel and suggested that sp^3^ fraction in the films becomes higher at lower deposition temperatures. Recently, Li et al. [20] investigated the effect of sputtering powers on the microstructure of DLC films on 316L stainless steel by using the direct current magnetron sputter technique. The obtained results showed that the sp^3^ content of DLC films increases with increasing sputtering power. These experiments clearly indicate that the microstructures of DLC films deposited on stainless steel substrates are quite sensitive to the depositing techniques and process parameters. This sensitivity opens plenty of opportunities to extrinsically tune the microstructures of DLC films for specific applications. However, a lack of understanding of the underlying mechanism of the formation of graphite–diamond interconnection in the DLC film make it difficult to find optimal control parameters to obtain desired microstructures.

Recently, molecular dynamics (MD) simulations have been used to investigate the growth of DLC films and have provided unprecedented looks at the microstructure evolution during the deposition process. In addition, the effects of various factors on structures and properties of DLC films have been clarified [21,22,23,24,25,26,27]. Most MD studies have simulated the deposition processes of DLC films on substrates composed of only one chemical composition. On the other hand, stainless steel substrates include several chemical compositions, such as Fe, Cr, and Ni, therefore, many pairwise interactions among Fe, Cr, Ni, and C atoms appear in the system. However, it is difficult to take into account these pairwise interactions in MD simulations, which require several corresponding potentials to be overcome simultaneously. Consequently, MD simulations for the growth processes of DLC films on stainless steel substrates have remained elusive.

In this study, the growth processes of DLC films on the AISI 316L stainless steel substrate were studied via the MD method. Several potentials were employed to describe the pairwise interactions among atoms of the substrate and the film. Effects of substrate bias voltage on the structure properties and sp^3^ ratio were investigated. In addition, the microstructure evolution of DLC film during the growth process was considered. Furthermore, we discuss the growth mechanism of DLC film on the stainless steel substrate.

## 2. Methods

### 2.1. Theoretical Framework

To simulate the deposition of DLC film on AISI 316L substrate, several potentials are used to include all possible interactions among atoms in the system. In the substrate, the embedded-atom method (EAM) potential [28] is used to describe pairwise interactions for Fe–Cr–Ni. In the thin film, the Tersoff potential [29] is adopted for C–C interactions. For the interaction between the film and the substrate, the Morse potential is used for C–Ni interactions, while the Tersoff/ZBL potential [30] is applied to (Fe, Cr)–C interactions.

Pairwise interactions of substrate atoms are adopted in the generalized form of EAM potentials due to Finnis and Sinclair [31]. The total energy *E_i_* of the *i*-th atom is given by

(1) $$E_{i}=F_{\alpha }(\sum_{j\neq i}\rho_{\alpha \beta }(r_{\mathrm{ij}}))+\frac{1}{2}\sum_{j\neq i}\phi_{\alpha \beta }(r_{\mathrm{ij}})$$

where *r_ij_* is the distance between atoms *i* and *j*; *α* and *β* refer the element types of atoms *i* and *j*; *F_α_* is the embedding function; *ϕ_αβ_* denotes a pair potential interaction; *ρ_αβ_* is the contribution of atom *j* of type *β* in the electron charge density at the site of atom *i* of type *α*.

The Tersoff potential is expressed as

(2) $$E=\frac{1}{2}\sum_{i}\sum_{j\neq i}V_{\mathrm{ij}}=\frac{1}{2}\sum_{i}\sum_{j\neq i}{\{f}_{C}(r_{\mathrm{ij}})[f_{R}(r_{\mathrm{ij}})+b_{\mathrm{ij}}f_{A}(r_{\mathrm{ij}})]\}$$

(3) $$f_{C}(r)=\left\{ \begin{array}{l} 1\\ \frac{1}{2}-\frac{1}{2}\sin(\frac{\pi }{2}\frac{r-R}{D})\\ 0 \end{array}\right.\;|\begin{array}{l} r<R-D\\ R-D<r<R+D\\ r>R+D \end{array}$$

(4) $$\begin{array}{l} f_{R}(r)=A\exp(-\lambda_{1}r)\\ f_{A}(r)=B\exp(-\lambda_{2}r) \end{array}$$

(5) $$b_{ij}={\left(1+\beta^{n}\xi_{ij}^{n}\right)}^{-\frac{1}{2n}}$$

(6) $$\xi_{ij}=\sum_{k\neq i,j}f_{C}(r_{ij})g(\theta_{ijk})\exp[\lambda_{3}^{m}{\left(r_{ij}-r_{ik}\right)}^{m}]$$

(7) $$g(\theta )=\gamma_{ijk}(1+\frac{c^{2}}{d^{2}}-\frac{c^{2}}{\left[d^{2}+(\cos\theta -\cos\theta_{0})\right]})$$

where *f_A_* and *f_R_* denote the attractive and repulsive pair potentials, respectively; *f_C_* represents the cut-off function; *b_ij_* is the bond-order term that represents the characteristic feature of the Tersoff potential; and *θ_ijk_* demotes the bond angle between bonds *ij* and *ik*.

The Tersoff/ZBL potential energy is given by [32].

(8) $$E=\frac{1}{2}\sum \sum_{j\neq i}V_{\mathrm{ij}}$$

(9) $$V_{ij}=(1-f_{F}(r_{ij}+\delta ))V_{ij}^{ZBL}+f_{F}(r_{ij}+\delta )V_{ij}^{Tersoff}$$

(10) $$f_{F}(r_{ij})=\frac{1}{1+e^{-A_{F}(r_{ij}-r_{C})}}$$

(11) $$V_{ij}^{ZBL}=\frac{1}{4\pi \varepsilon_{0}}\frac{Z_{i}Z_{j}e^{2}}{r_{ij}}\phi (r_{ij}/a)+S(r_{\mathrm{ij}})$$

(12) $$a=\frac{0.8854a_{0}}{Z_{i}^{0.23}+Z_{j}^{0.23}}$$

(13) $$\phi (x)=0.1818e^{-3.2x}+0.5099e^{-0.9423x}+0.2802e^{-0.4029x}+0.02817e^{-0.2016x}$$

where, *f_F_* denotes the fermi-like function; *A_F_* is the control parameter; *r_C_* is the cut-off radius for the ZBL potential; *Z_i_* and *Z_j_* are the number of protons in each nucleus; *e* is the electron charge; *ε*_0_ is the permittivity of vacuum; *a*_0_ is the Bohr radius.

All pairwise interactions for Fe–Cr–Ni, Fe–C, and C–C are described by potential energies integrated in the large scale atomic/molecular massively parallel simulator (LAMMPS), excepted for C–Cr and C–Ni interactions. Based on Henriksson’s calculations for the C–Cr pair [30], we transformed the pair coefficients of the Analytic Bond-Order Potential (ABOP) formula to the Tersoff/ZBL implementation in LAMMPS using the following formulation: *m* = *n* = 1, *β* = *ω*, $\lambda_{1}=\beta \sqrt{2S}$, $\lambda_{2}=\beta \sqrt{2/S}$, λ_3_ = α_ijk_, cos*θ*_0_ = −h, $A=\frac{D_{0}}{S-1}e^{-\lambda_{1}r_{0}}$, $B=\frac{SD_{0}}{S-1}e^{-\lambda_{2}r_{0}}$. The parameter sets used in this study are compiled in Table 1.

Morse potential is expressed as

(14) $$E=D_{0}\left[e^{-2\alpha \left(r-r_{0}\right)}-2e^{-\alpha \left(r-r_{0}\right)}\right]\;r\;<\;r_{c}$$

where *r* is the distance between the atoms; *r*_0_ is the equilibrium bond distance; *α* controls the “width” of the potential; *D*_0_ is the well depth. We have determined the parameters of the Morse potential (*D*_0_ = 2.431 eV, *α* = 3.295 Å-1, *r*_0_ = 1.763 Å, and *r_c_* = 11.58 Å) by fitting the C–Ni interaction obtained in the previous study [33].

### 2.2. Model Establishment

**Figure 1:**
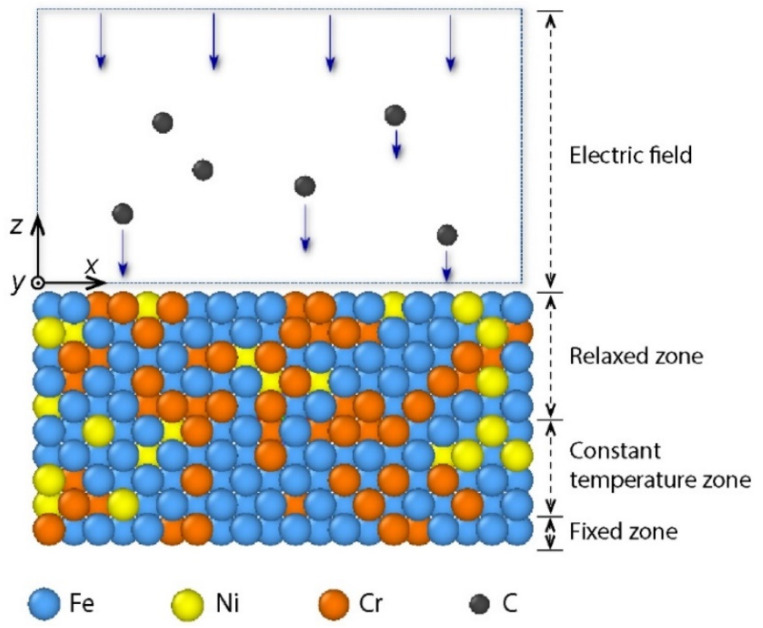
Schematic illustration of carbon film growth on AISI 316L substrate.

A schematic illustration of DLC film growth on AISI 316L stainless steel substrate is represented in Figure 1. The AISI 316L substrate that was constructed with a face-centered cubic structure was composed of 1386 Fe atoms, 360 Cr atoms, and 246 Ni atoms. The mole fraction ratio of Fe:Cr:Ni was 69.6:18.1:12.3. The substrate dimensions were 36 Å × 36 Å × 15.6 Å. To mimic a real experimental situation, the substrate atoms were divided into three regions, including a fixed zone, a constant temperature zone, and a relaxed zone. One atomic layer at the bottom of substrate was assigned as the fixed zone, in which the positions of atoms did not change during the deposition process. Four atomic layers in the middle of substrate were included in the constant temperature zone, where the positions of atoms could change during the deposition process, while the temperature was maintained around 300 K [34]. Five atomic layers at the top of substrate were used in the relaxed zone, where the position and temperature of atoms could change with the deposition process. A vacuum layer of 40 Å was arranged above the substrate. The substrate bias voltage *V_S_* was considered in a wide range from 0 V to 300 V. In the MD simulation, an electric field equivalent to the bias voltage was applied perpendicular to the substrate during the deposition. Note that the surface of stainless steel is commonly covered by an oxide layer. However, in the previous experiment [17], the oxide layer on the surface of the stainless steel substrate was removed before the carbon film was deposited. In addition, the chamber of the closed unbalanced magnetron sputtering system was pumped below 3×10^−5^ torr, such that the residual oxygen in the chamber was negligible. Therefore, in this study, the MD model of the stainless steel substrate without an oxide layer was relevant to the experiment [17].

### 2.3. Simulation Process

Large-scale atomic/molecular massively parallel simulator (LAMMPS) code [35] was employed to simulate the growth process of DLC film on the AISI 316L substrate. Periodic boundary conditions were applied to x and y directions. Tersoff/zbl and Mose potentials [30] were used to control the interactions between C atoms of the deposition and Fe, Cr, and Ni atoms of the substrate. The simulation time step was 1.0 fs. Three thousand C-atoms were incident perpendicularly to the substrate from 36 to 38 Å above the substrate. Previous studies have suggested that the incident kinetic energy of deposited particles in magnetron sputtering is in a range from 10 to 30 eV [36,37,38], therefore, the incident energy was selected as 15 eV/atom in this study. The time interval between two incident atoms was 0.1 ps.

## 3. Results and Discussion

### 3.1. Deposition Process of DLC Thin Films

**Figure 2:**
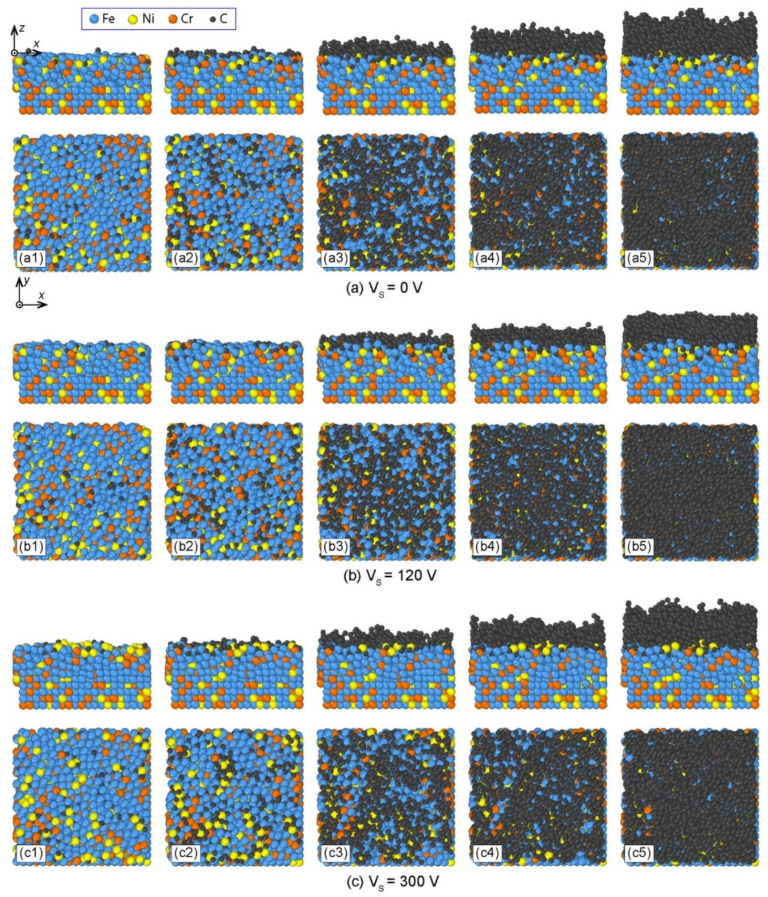
Deposition processes of carbon thin films on AISI 316 stainless steel substrates under different substrate bias voltages: (**a**) *Vs* = 0 V, (**b**) *Vs* = 120 V, and (**c**) *Vs* = 300 V.

The deposition process of DLC film under 0 V bias voltage is represented in Figure 2a, in which the front and top views of deposition structures were placed at the upper and lower parts of the figure, respectively. At the initial stage of deposition, the incident C atoms collided with substrate atoms, i.e., Fe, Ni, and Cr atoms. Since the C atoms have small diameters and obtained high energy, they could diffuse into the interstices among Fe, Cr, and Ni atoms in the substrate, as shown in Figure 2(a1). When the atomic interstices in the surface layer of AISI 316L substrate became saturated, the subsequent incident C atoms could stabilize and form protruding island-like structures on the substrate surface (Figure 2(a2)). The initial surface structure of the substrate was then disturbed due to the constant impact of high energy C atoms. At this stage, the C atoms could displace the original positions of Fe, Cr, and Ni atoms in the surface layer of the substrate. As a result, a compositional transition layer, which mixed Fe, Cr, Ni, and C atoms, was formed between the C film and the AISI 316L substrate (Figure 2(a3)). Meanwhile, as the island-like structures of C film gradually extended to surrounding regions, they could connect with each other to form a network structure. Afterward, the subsequent incident C atoms only collided with the deposited C atoms and were intact by substrate atoms. The C film gradually grew layer by layer until the final deposition structure of C film was achieved, as shown in Figure 2(a4,a5). The deposition process of C film on AISI 316L substrate under 0 V bias voltage could be divided into four stages, namely, the C-atom diffusion stage, island-formation stage, substrate-atoms diffusion stage, and stable growth stage.

Similarly, the deposition processes of C films with bias voltages of 120 V and 300 V are shown in Figure 2b,c, respectively. The four stages of the deposition process were also observed for different bias voltages. However, when the bias voltage increased, C atoms achieved higher energy, and thereby could penetrate deeper into the AISI 316L substrate in the C-atom diffusion stage, as shown in Figure 2(b1,c1). In addition, the substrate–film diffusion layer changed with the bias voltage. Therefore, the substrate bias voltage affected the deposition process of C film on the AISI 316L substrate.

### 3.2. Microstructure Characterization

**Figure 3:**
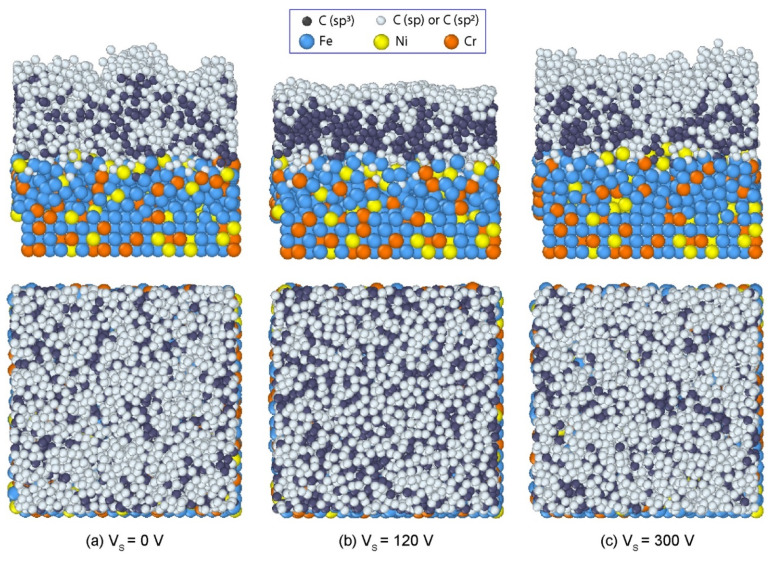
Structures of DLC films formed on the AISI 316L substrate with different bias voltages: (**a**) 0 V, (**b**) 120 V, and (**c**) 300 V.

In Figure 3, the final structures of C films with the representative bias voltages of 0 V, 120 V, and 300 V are shown. The whole system can be divided into four regions including substrate, transition, stable, and surface regions. At 0 V bias voltage (Figure 3a), C film with high roughness was generated. In the substrate region, a small amount of C atoms diffused into the interstices among Fe, Cr, and Ni atoms in the substrate. The incident atoms had a small effect on the original arrangement of substrate atoms. When the bias voltage was 120 V, the film achieved a compact structure with a smooth surface, as shown in Figure 3b. The film was thinner than that with zero bias voltage. Since the incident energy increased when the bias voltage increased, the incident C atoms could implant deeper into the AISI 316L substrate, resulting in a larger intermixing layer at the interface. When the bias voltage was 300 V, an incompact structure of C film was formed again (Figure 3c). The film surface exhibited a high roughness. Therefore, the substrate bias voltage significantly affected the structures and morphologies of deposited films, in which the compact structure with a smooth surface could be formed as *Vs* = 120 V, while looser structures with rough surfaces appeared as *Vs* = 0 or 300 V.

**Figure 4:**
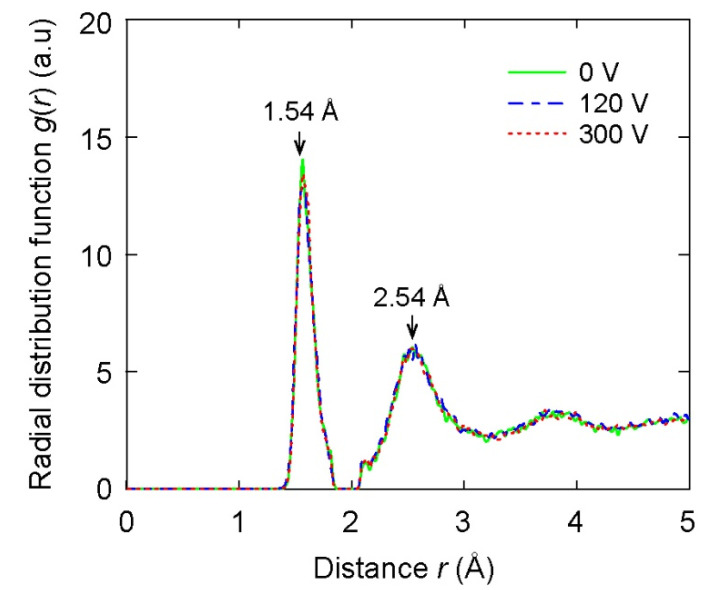
The radial distribution function of the DLC films according to different substrate voltage values.

Given the structures of C films, the radial distribution functions (RDF) of films are represented for different bias voltages, as shown in Figure 4. All considered films exhibited similar RDF patterns. Importantly, there were two notable peaks in each RDF pattern, as shown in Figure 4. The first and second peaks in the RDF of C films were located near 1.54 Å and 2.54 Å, respectively. In a previous study [26], the first and second peaks in the RDF of diamond also appeared at 1.54 Å and 2.54 Å, respectively. Hence, the first and second peaks in the RDF of C films formed with different bias voltages were observed at similar positions in comparison to those of diamond. This result shows that the C films include similar structures of diamonds. In addition, the RDF shown in Figure 4 exhibited that all films were amorphous with the characteristics of long-range disorder and short-range order, representing DLC structures. Notably, there was a small peak at the position of 2.1 Å shown in Figure 4. The origin of this small peak was previously attributed to the cut-off radius of the Tersoff potential [39,40].

**Figure 5:**
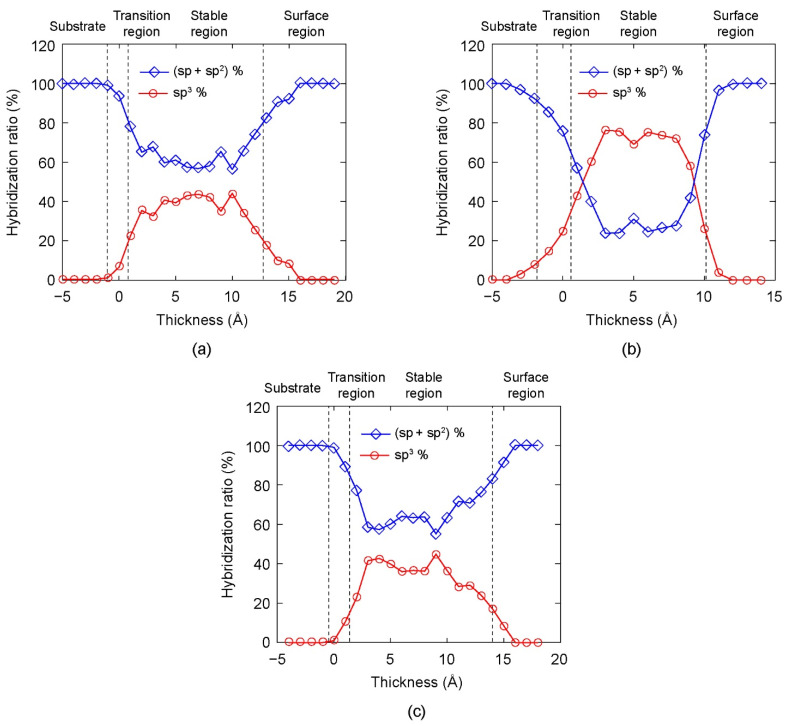
The hybridization ratio along the thickness direction of DLC film under different substrate bias voltages: (**a**) 0 V, (**b**) 120 V, and (**c**) 300 V.

The variations of the hybridization ratio in the DLC films along the film growth direction are plotted in Figure 5 for different bias voltages. The horizontal axis represents the thickness of DLC film, where the origin of the horizontal axis is selected at the surface of the initial AISI 316L substrate. The vertical axis represents the hybridization ratio of C atoms. The variation of the hybridization ratio along the DLC film thickness is shown in Figure 5a when the substrate bias voltage was 0 V. In the transition region, large numbers of Fe, Cr, Ni, and C atoms mixed, therefore it was relatively difficult to form sp^3^–C bonds. As a result, the percentage of sp^3^–C atoms was low. In the stable region, the percentage of sp^3^–C atoms increased. There was a small fluctuation in the percentage of sp^3^–C in the stable region. In the surface region, the percentages of sp^3^–C atoms in the DLC film sharply decreased to zero. Therefore, a high percentage of sp^3^–C atoms concentrated in the middle of DLC film. Similar tendencies of the hybridization ratio variation were observed in DLC films deposited under higher bias voltages (Figure 5b,c). However, the magnitude of sp^3^ percentage changed according to the bias voltage. This result showed that the hybridization ratio significantly depended on the substrate bias voltage.

**Figure 6:**
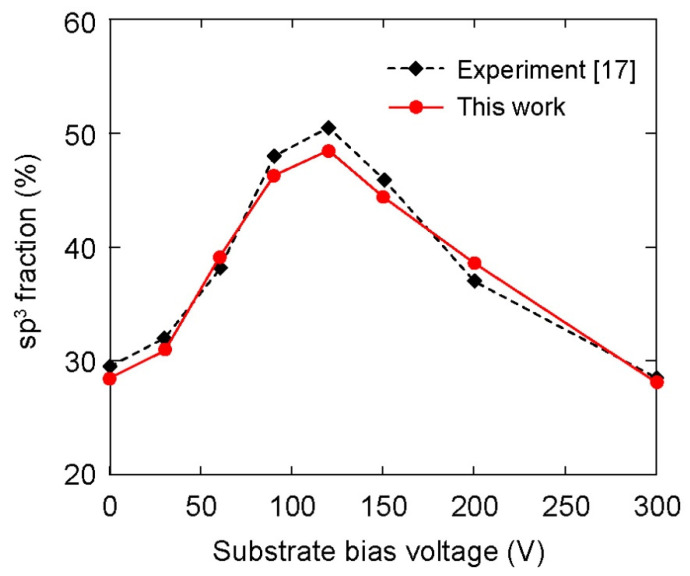
Effect of substrate bias voltage on overall sp^3^ fraction in the DLC films. The experimental results were adopted from the previous study [17].

The dependence of the sp^3^–C hybridization ratio on the substrate bias voltage is shown in Figure 6. The variation of sp^3^ fraction was like a parabola versus the substrate bias voltage in a range from 0 V to 300 V. At 0 V bias voltage, sp^3^ fraction achieved a magnitude of about 28.5%. Sp^3^ fraction first increased with the increase in substrate bias voltage from 0 V to 120 V; however, an opposite trend was observed when the bias voltage was further increased from 120 V to 300 V. The highest magnitude of sp^3^ fraction was about 48.5% at 120 V bias voltage. For comparison purposes, the variation of the sp^3^ ratio on the bias voltage that was obtained from experiment for the deposition of DLC film on the AISI 316L substrate [17] is also included in Figure 6. Obviously, MD simulation results exhibited small differences at all considered bias voltages in comparison to the experimental ones. This consistency, otherwise, validated the present simulation model. Note that the validity of the comparison in Figure 6 stems from the hypothesis that the substrate was assumed oxide-free.

### 3.3. Discussion

Here, we discuss a potential mechanism for the dependence of sp^3^ fraction and DLC film structure on the substrate bias voltage. When the bias voltage was small (<50 V), the incident energy was low and the deposited C atoms were randomly absorbed in the low energetic positions of the AISI 316L substrate. With the further deposition of C atoms, the incident atoms tended to stick on top of the pre-deposited atoms, and thereby form a loosely packed graphite-like (sp^2^) structure. Due to the adsorption of incident atoms, sp^3^ fraction was much lower than sp and sp^2^ fractions (Figure 5a) and many defects existed in this film (Figure 3a). When the bias voltage increased from 50 V to 120 V, the incident C atoms achieved relatively higher energy. There are two phenomena that can simultaneously occur as high energy C atoms approach the pre-deposited surface, namely, subplantation [41,42] and pre-existing surface burial [43]. In the former phenomenon, the incident atoms penetrate the deposited film and enter interstitial or porous positions. Accumulation of these atoms increases the local density. In the latter phenomenon, the newly deposited atoms locate in surface sites (i.e., bury pre-existing surface) and impose compression to the deposited film due to the high incident energy, leading to the collapse and densification of the porous structure. Both phenomena result in a high density and compact film. Such a high-density film with compression leads to a reformation of local bonding around the C atoms with appropriate hybridization. On the other hand, density functional theory calculations indicate that the activation barrier for graphite–diamond interconversion is 0.33 eV/atom [44]. The bias voltage larger than 50 V provided sufficient energy for the sp^2^-to-sp^3^ transformation. As a result, sp^3^ fraction in DLC film increased with increasing bias voltage as Vs ≤ 120 V. When the bias voltage further increased (>120 V), the intense C atomic bombardment could break the formed sp^3^ structure, and, consequently, sp^3^ fraction was reduced. Such a damage mechanism that reduced sp^3^ fraction due to impact incident atoms with high energy was also discussed in previous studies [26,45,46]. In addition, the deposited C atoms could obtain energy from the incident atoms by colliding and escaping from the equilibrium position, giving rise a looser structure of film when the bias voltage was too large (Figure 3c).

In this study, the highest sp^3^ fraction (48.5%) in DLC film deposited on the AISI 316L substrate was about two times larger than that deposited on γ-Fe substrate (26.2%) [25]. This result can be explained from the interactions between the incident C atoms and the substrate. Since the Fe–Cr and Fe–Ni biding energies were larger than that of Fe–Fe [47,48,49], the incident C atoms disturbed the γ-Fe substrate more severely than the AISI 316L substrate. Consequently, the transition region in DLC film on the γ-Fe substrate was larger than that on the AISI 316L substrate. In turn, the large transition region hindered the formation of sp^3^ hybridized bonds in the DLC film on the γ-Fe substrate. On the other hand, the highest sp^3^ fraction in DLC film deposited on the AISI 316L substrate in this study was comparable to that deposited on Si or glass substrates [46,50,51].

In addition, the formation of a surface region with a sharp decrease of sp^3^ content toward the film surface in all simulation cases (as shown in Figure 5) agreed well with previous experimental and simulation studies [25,26,27,52,53]. Such a formation of a graphite-like surface layer is attributed to less atomic bombardment than the stable region and surface relaxation. Specifically, during surface relaxation, the C atoms near the film surface shifted to lower energy sites, facilitating the formation of sp^2^ hybridized bonds. In addition, the decrease of compressive stress during surface relaxation promoted rehybridization from sp^3^ to sp^2^ bonds [46].

The DLC film was composed mainly of C atoms with sp^3^ and sp^2^ hybridizations, forming σ (sp^3^) and π (sp^2^) bonds, where σ bonds determined the mechanical properties and π bonds affected the electrical and optical conductance. Generally, large sp^3^ fraction in DLC film enhances the mechanical properties. Therefore, control of the sp^3^/sp^2^ carbon ratio can achieve a wide range of properties. In this study, the bias-induced change of sp^3^ fraction in a wide range from 28.5% to 48.5% (as shown in Figure 6) suggests an effective approach to control the sp^3^/sp^2^ ratio, which appears to be promising for various applications. For example, in medical applications, compact DLC films with high sp^3^ fraction coating on stainless steel substrates can prevent the corrosion and release of Cr, Ni, Mn, and Mo ions when the metal is placed in coronary vessels, and thereby increase the biocompatibility of the implant materials and suppress allergic reactions [54]. In addition, high sp^3^ fraction in DLC films enhances the corrosion resistance and hardness of the film, which can be used as a protective coating in the industrial fields of cutting tools and molds [19,55,56]. On the other hand, applications of electrode materials and bipolar plates commonly require sufficient electrical conductivity and high corrosion resistance. A proper control of sp^3^/sp^2^ ratio in DLC film can balance both requirements, since a change of sp^3^/sp^2^ can also tune the electrical resistivity of the DLC film in a wide range from 10^12^ to 10^16^ Ωcm [11,57].

## 4. Conclusions

Deposition of DLC thin films on the AISI 316L stainless steel substrate and their structural properties were investigated under different substrate bias voltages by using molecular dynamics simulations. Different potentials were used for the pairwise interactions among Fe, Cr, Ni, and C atoms. The structure of DLC films after deposition could be divided into four regions, including the substrate, transition, stable, and surface regions. There were a few C atoms in the substrate region. In the transition region, C atoms of the deposition mixed with the Fe, Cr, and Ni atoms of the substrate. In the stable region, large numbers of C atoms formed sp^3^ hybridized bonds. In the surface region, C atom bonds were relatively loose, and were mainly sp and sp^2^ hybridized bonds. The overall sp^3^ fraction in DLC films increased with the increase of substrate bias voltage from 0 V to 120 V; however, an opposite trend was observed when the bias voltage is further increased from 120 V to 300 V. The highest magnitude of sp^3^ fraction was about 48.5% at 120 V bias voltage. Furthermore, low bias voltages gave rise to a loose structure of DLC film with several defects. Sufficient large bias voltages facilitated the formation of compact structures with high sp^3^ fraction, while too large a voltage can break the formed sp^3^ structure, giving rise to a loose structure.

## Figures and Tables

**Table 1:** Parameters of Tersoff and Tersoff/ZBL potentials used in MD simulations [29,30].

Parameters	C–C [29]	C–Fe [30]	C–Cr [30]
*m*	3	1	1
*γ*	1	0.074856	0.000688
*λ*_3_	0	0	0.612216
*A* (eV)	1544.8	1035.475541	0.001679
*B* (eV)	389.63	357.343176	0.148484
*λ*_1_ (Å^−1^)	3.4653	3.080134	4.062626
*λ*_2_ (Å^−1^)	2.3064	2.153411	1.985278
*β* (Å^−1^)	4.1612 × 10^−6^	1	0.294
*n*	0.99054	1	1
*c*	19981	1.116742	3.933538
*d*	7.034	0.946631	0.174972
cos*θ*_0_	−0.33953	−0.186653	−0.1785
*R* (Å)	1.95	2.6	2.95
*D* (Å)	0.15	0.2	0.1
*A_F_* (Å^−1^)	-	10	8
*r_C_* (Å)	-	1.0	1.2

## Data Availability

The data presented in this study are available on request from the corresponding author.
